# Massive Hematemesis from a Splenic Artery Pseudoaneurysm Presenting Two Years after Penetrating Trauma

**DOI:** 10.1155/2018/7473168

**Published:** 2018-05-13

**Authors:** Geraldine Abbey-Mensah, Michael M. Herskowitz, James Walsh, Robert F. Leonardo

**Affiliations:** ^1^Kings County Hospital Center, Department of Radiology, 451 Clarkson Ave., Brooklyn, NY 11203, USA; ^2^South Nassau Communities Hospital, Department of Radiology, Oceanside, NY 11572, USA

## Abstract

Splenic artery pseudoaneurysms (PSA) are rare entities and far less common than true aneurysms of the splenic artery. The most common etiology is pancreatitis, recurrent either in the setting of chronic pancreatitis or as an episode of acute pancreatitis. Less common causes include trauma, peptic ulcer disease, or iatrogenic causes. Almost all of the trauma-related case reports have been due to blunt trauma. We believe this to be the first reported case of a splenic artery PSA presenting with massive hematemesis at a significant time frame after penetrating trauma. Successful transcatheter treatment was performed and alternative techniques are also discussed.

## 1. Introduction

Splenic artery pseudoaneurysms (PSA) are far less common than true aneurysms of the splenic artery. They are predominately caused by pancreatitis, either recurrent or acute, and less commonly caused by trauma, peptic ulcer disease, or iatrogenic causes. They have a higher incidence of rupture and significant morbidity when discovered as compared to true aneurysms. We present an unusual case of a splenic artery PSA presenting with massive hematemesis approximately two years after penetrating trauma of the upper abdomen. It was successfully treated with transcatheter embolization which is now accepted as the preferred approach to such cases, compared with more invasive surgical approaches.

## 2. Case Report

A 22-year-old-male presented to the emergency department with a one day history of abdominal pain, syncope, and three episodes of hematemesis. The patient denied any previous history of abdominal pain, gastrointestinal bleeding, or altered bowel habits. The patient had suffered multiple deep stab wounds to the left upper abdomen approximately two years earlier, resulting in emergency laparotomy, transverse colostomy, and an uneventful colostomy reversal two months later. He admitted to heavy weekend alcohol use, occasional recreational drug use, and smoking one pack of cigarettes per day for six years. There was nothing in the history to suggest any bouts of pancreatitis or gastric ulcer disease.

In the emergency department the patient was hypotensive to 100/50 with a pulse of 90. Laboratory examination showed hemoglobin = 8.8 g/dL (normal range 12.0–16.0 g/dL), hematocrit = 26.5% (normal range 37.0–47.0%), platelets = 195 k/uL (normal range 130–400 K/uL), and WBC = 20.2 K/uL (normal range 3.50–10.80 K/uL). All other values including coagulation profile were within normal limits.

It was decided to proceed directly to gastroenterology (GI) for upper endoscopy, which revealed a large adherent clot at the mid greater curvature of the stomach. There was no evidence for ulceration at the site of the adherent clot and a biopsy was not performed. There was fresh blood in the antrum and duodenum, consistent with recent bleed. No active bleeding site could be seen from the antrum or duodenum and no blood was seen coursing from the ampulla. It was determined that the mid greater curvature site in the body of the stomach most likely represented the source of the hematemesis.

**Figure 1:**
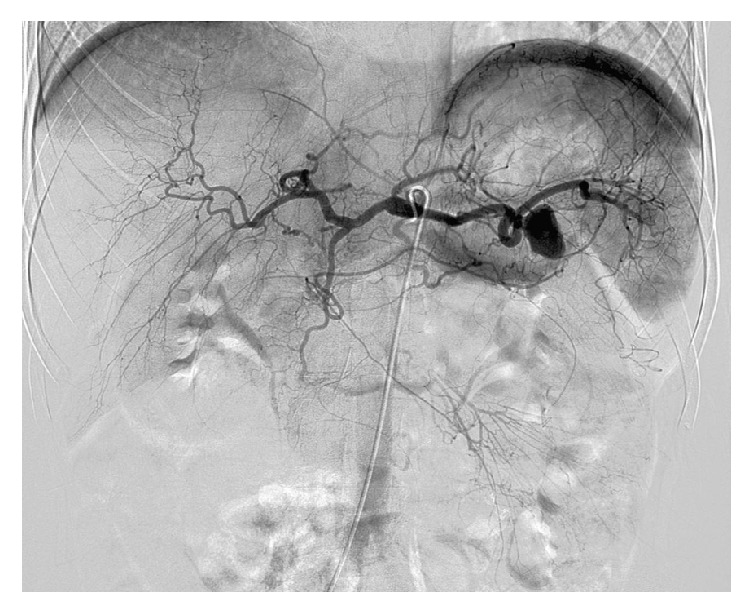
Celiac axis arteriogram demonstrating a large pseudoaneurysm from the mid-to-distal splenic artery.

**Figure 2:**
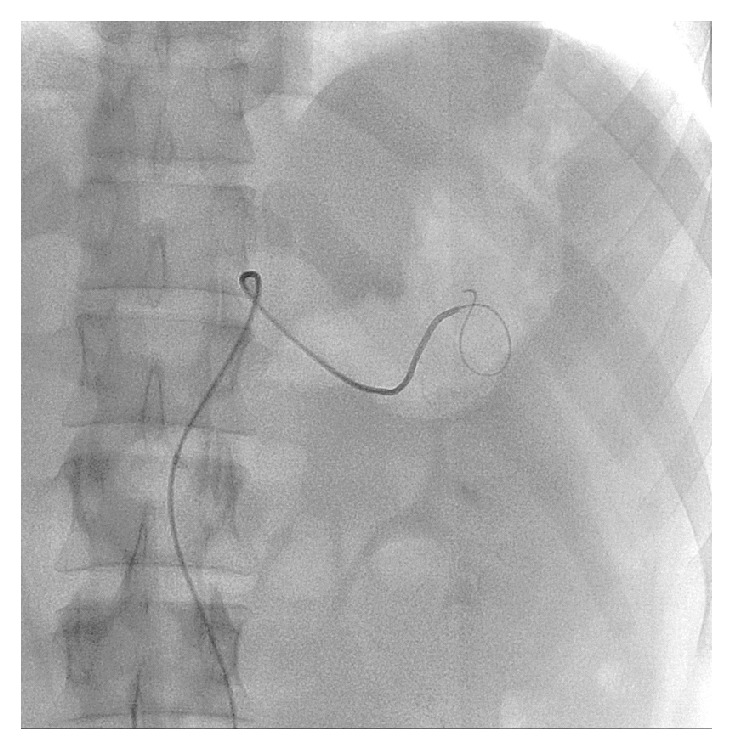
Angiographic catheter cannulating the pseudoaneurysm for coil packing.

**Figure 3:**
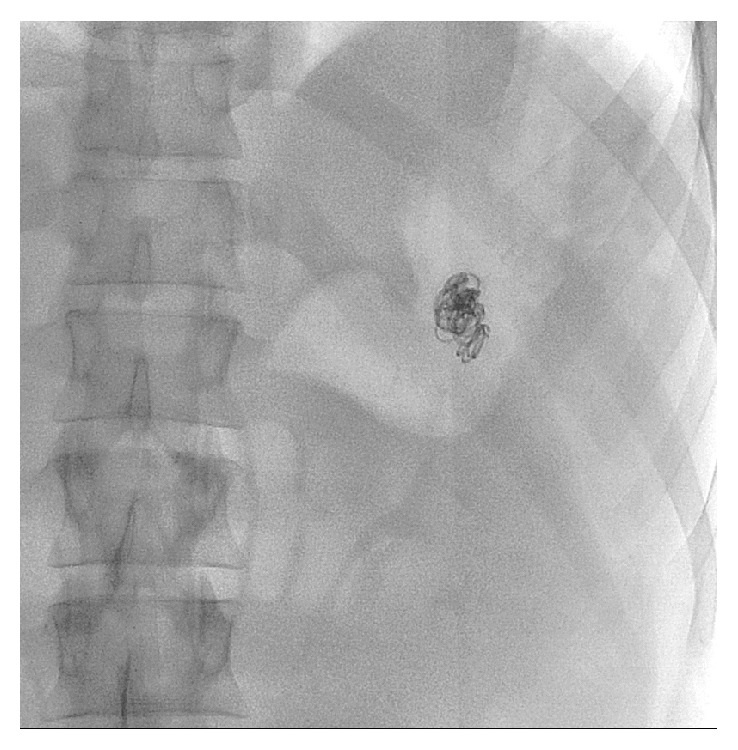
Final image of dense coil packing and thrombosis of the pseudoaneurysm.

Based on the presentation of hematemesis and hypotension, the gastroenterologists recommended emergent angiography with deferment of CT imaging. Celiac and splenic arteriograms demonstrated a large wide-necked PSA from the mid-to-distal splenic artery with no active extravasation (Figure 1). It was first attempted to perform proximal and distal coil embolization of the splenic artery to exclude the PSA, but due to extreme tortuosity, neither a catheter nor a coaxial wire could be advanced distal to the PSA. The 5-French angiographic catheter was then advanced into the PSA (Figure 2) and the PSA was tightly packed with eight 8 mm diameter and six 5 mm diameter Tornado metallic coils (Cook, Inc. Bloomington, IN) (Figure 3). Postembolization arteriogram showed compete occlusion of the PSA and normal preserved flow into the spleen.

The patient tolerated the procedure well with no immediate complications. The postprocedure course was uneventful as the blood pressure and hemoglobin normalized and there were no further episodes of gastrointestinal bleed reported on a return to GI clinic in 10 days. The patient was subsequently lost to followup.

## 3. Discussion

Splenic artery pseudoaneurysms (PSA) are rare entities and far less common than true aneurysms of the same vessel [1–5]. They most commonly occur as a complication of pancreatitis, either recurrent or less commonly a new bout of acute pancreatitis. It is stated that up to 10% of patients with pancreatitis develop arterial complications as a result of activated pancreatic enzymes digesting the arterial wall. The splenic artery is the most common of these involved vessels. Less common causes include abdominal trauma, peptic ulcer disease, and iatrogenic causes. Abdominal trauma as a cause of splenic PSA is usually blunt rather than penetrating and more often intrasplenic rather than isolated to the main splenic artery [3–5]. The standard imaging evaluation for all visceral pseudoaneurysms would be contrast enhanced CT scan. However, our patient was deemed to be unstable based on hematemesis and hypotension and was taken directly to angiography.

The risk of rupture is greater for splenic artery PSA compared to true aneurysms and the resultant morbidity and mortality are significant. If they are incidentally discovered it is generally accepted that prompt treatment should be instituted. Traditional open surgery either splenectomy with or without distal pancreatectomy or splenic artery ligation and PSA resection were the main forms of treatment. Over the past three decades, endovascular therapy has become the accepted and preferred treatment modality in hemodynamically stable patients.

The preferred technique for endovascular treatment of splenic PSA is proximal and distal coil embolization as close as possible to the PSA [4–8]. This so called “sandwich technique” will result in thrombosis of the PSA and preserve splenic arterial flow and function via collaterals from the left gastric, gastroepiploic and transverse pancreatic vessels into the short gastric vessels and back into the splenic hilum. This method was not technically feasible in our case.

Dense coil sac packing was used in our case. This method spares flow in the entire parent vessel but may be technically more challenging. Another endovascular treatment option is placement of a covered stent-graft [9]. However, the rigidity of such systems precludes the ability to safely place such grafts far into the splenic artery in most cases. A further option is percutaneous fine needle access to the PSA followed by thrombin injection with or without coil placement [10], which should only be considered a method of last resort if all transcatheter treatments have been considered and deemed not possible.

As mentioned, our patient was a relatively healthy young male who suffered penetrating trauma to the left upper abdomen two years prior to this admission. Although there was a history of alcohol use, there were no other hospital admissions and no history of abdominal pain to suggest that pancreatitis had ever occurred. Likewise a gastric ulcer as the inciting event is highly unlikely based on the history and the lack of chronic abdominal pain. The gastroenterologist did not request a stool examination for* H. pylori* or perform biopsy of the stomach, and the appearance at endoscopy tended to rule out peptic ulcer disease. The splenic artery PSA in all likelihood developed at the time of the initial trauma was not recognized at the time of laparotomy or colostomy reversal and subsequently eroded into the body of the stomach.

The length of time from injury to presentation of two years is highly unusual. Most delays from injury to presentation have ranged from one day to four months. Patients suffering penetrating abdominal trauma requiring urgent laparotomy often do not undergo routine CT scans, which may have led to an earlier diagnosis before the complication of hematemesis in this case. It can be argued that all patients suffering significant penetrating abdominal trauma undergo CT scan of the abdomen and pelvis in attempt to rule out unsuspected vascular complication. This can be performed either prior to or after laparotomy, depending on the clinical circumstances.

Several authors have described delayed presentations of splenic artery pseudoaneurysms, but these have always been related to blunt trauma, and usually intrasplenic, rather than localized to the splenic artery proximal to the spleen [11, 12]. We believe that this is the world's first reported case of a splenic artery PSA from penetrating trauma causing massive hematemesis and presenting after a significant time interval from the initial event.
